# Infection model of THP-1 cells, growth dynamics, and antimicrobial susceptibility of clinical *Mycobacterium abscessus* isolates from cystic fibrosis patients: Results from a multicentre study

**DOI:** 10.1371/journal.pone.0319710

**Published:** 2025-03-31

**Authors:** Alba Ruedas-López, Marta Tato, Laura Lerma, Jaime Esteban, María-Carmen Muñoz-Egea, Carlos Toro, Diego Domingo, Rafael Prados-Rosales, Paula López-Roa

**Affiliations:** 1 Clinical Microbiology and Parasitology Department, Hospital Universitario 12 de Octubre, Madrid, Spain; 2 Department of Preventive Medicine, Public Health and Microbiology, School of Medicine, Universidad Autónoma de Madrid, Madrid, Spain; 3 Clinical Microbiology and Parasitology Department, Hospital Universitario Ramón y Cajal, Madrid, Spain; 4 .Clinical Microbiology Department, IIS-Fundación Jiménez Díaz, UAM. Madrid, Spain; 5 CIBERINFEC-CIBER de Enfermedades Infecciosas, Madrid, Spain; 6 Clinical Microbiology and Parasitology Department, Hospital Universitario La Paz, Madrid, Spain; 7 Clinical Microbiology and Parasitology Department, Hospital Universitario de La Princesa, Madrid, Spain; The University of New Mexico School of Medicine, UNITED STATES OF AMERICA

## Abstract

*Mycobacterium abscessus* (MABS) is an emerging pathogen causing severe infections, particularly in cystic fibrosis (CF) patients. A prospective multicentre study included CF patients from four hospitals in Madrid between January 2022 and January 2024. Respiratory samples were collected, and MABS isolates were analysed to determine their antibiotic resistance profiles, growth dynamics, infection kinetics, intracellular behaviour, and pathogenicity. Intracellular bacterial growth and macrophage viability were evaluated through THP-1 cell infection experiments, with and without amikacin. Phenotypic susceptibility testing and genotypic susceptibility testing were also conducted. Among 148 patients, 28 MABS isolates were detected from 16 patients (10.8%), and the first isolate from each patient was analysed. Isolation was more prevalent in younger individuals (median age 24.4 vs. 28.4 years, p = 0.049), and most isolates (81.25%) were identified as *M. abscessus subsp. abscessus* (MABSa)*.* MABS isolates exhibited high resistance rates (>85%) to doxycycline, tobramycin, ciprofloxacin, moxifloxacin (75%) and cotrimoxazole (56.3%). Amikacin resistance (18.8%) was higher than expected, and inducible (10/16 isolates) or acquired (1/16 isolate) macrolide resistance was found in 68.8% of strains. Phenotypic and genotypic testing results were fully concordant. Tigecycline demonstrated strong *in vitro* activity, and resistance to imipenem, linezolid, and cefoxitin remained low. Rough strains displayed lower optical density values in later growth stages, probably due to their increased aggregation. In THP-1 cell infection experiments, rough strains showed higher intracellular bacterial loads with statistically significant differences observed at 2 hours (both with and without amikacin) and at 72 hours (with amikacin) post infection. Notably, rough strains also exhibited a higher internalisation index and greater impact on THP-1 cell viability, especially in the absence of amikacin.

## Introduction

*Mycobacterium abscessus* (MABS) is a rapidly growing non-tuberculous Mycobacteria (RG-NTM) responsible for soft tissue [1] and wound infections [2], as well as progressive pulmonary diseases [3,4]. MABS has increasingly been recognised as an emerging pathogen, especially in patients with chronic lung conditions such as Cystic Fibrosis (CF) [5,6]. This species is classified into three subspecies: *M. abscessus subsp. abscessus* (MABSa), *M. abscessus subsp. massiliense* (MABSm) and *M. abscessus subsp. bolletii* (MABSb) [7]. Clinically, this distinction is crucial, as MABSa and MABSb typically show inducible macrolide resistance due to the *erm*(41) gene, whereas MABSm, with a truncated *erm*(41), remains susceptible to macrolides [8] in the absence of other resistance mechanisms. Furthermore, MABS is able to promote immune evasion, persistent infections [9–11], and resistance to diverse antibiotics [12], representing a complex challenge in the treatment of CF patients [6]. MABS strains exhibit distinct colony morphologies when cultured on solid media. Rough (R) colonies are characterised by a dry and irregular surface with numerous crests and folds, while smooth (S) colonies display a uniform, shiny, and moisty appearance [13,14]. The R morphotype, which lacks glycopeptidolipids (GPL) in its cell wall, is associated with pronounced bacterial clumping and the formation of cord-like structures [15]. This feature has been linked to increased virulence in *in vitro* studies [13], animal models [13,16] and humans [17]. The aggregation phenomenon complicates the accurate measurement of optical density (OD) and its correlation with colony-forming units (CFU). This challenge is significant as the CFU-OD relationship is vital for preparing bacterial inocula of known concentration for subsequent *in vitro* experiments.

THP-1 cells, derived from human monocytic leukaemia [18], serve as a model to study macrophage responses to infections. Previous studies have shown that MABS isolates with high virulence in other models, such as the silkworm infection model, also exhibit increased cytotoxicity to human THP-1-derived macrophages [19]. Differentiated THP-1 cells mimic human macrophages, providing a valuable system for investigating the intracellular behaviour, virulence and intracellular drug testing of pathogens such as MABS [20,21].

Here, we sought to characterise the growth dynamics, virulence, and antimicrobial susceptibility of MABS clinical strains. We aimed to investigate potential differences in the infection kinetics of these clinical MABS strains within THP-1 cells by evaluating their internalisation and proliferation capacities and their ability to impact THP-1 cell viability. Additionally, we sought to optimise and identify the limitations of the methodologies employed, including the assessment of the impact of amikacin on infection experiments.

## Materials and methods

### Ethics statement

This study was conducted following the ethical principles of the Declaration of Helsinki and received approval from the Institutional Ethics Committee of Hospital 12 de Octubre (reference number: CEIm 21/592), ensuring compliance with international ethical standards. All procedures adhered to hospital biosafety regulations and were approved by the Local Institutional Review Board. Written informed consent was obtained from all participants.

### Study design and data collection

Following approval from our Local Institutional Review Board, we conducted a prospective multicentre study including patients from four tertiary hospitals with CF units in Madrid. The recruitment period extended from January 1, 2022, to April 30, 2023. The study protocol entailed an initial baseline visit (month 0), followed by three visits at three-month intervals (months 3, 6, and 9), an annual visit (month 12), and a final visit at two years, which has not yet been completed for most patients. During the follow-up visits, respiratory samples were obtained, and clinical and microbiological data were collected and managed using REDCap (Research Electronic Data Capture) tools [22]. The bacterial strains included in the study were those isolated during the visits conducted between January 1, 2022, and January 1, 2024.

### Phenotypic and genotypic susceptibility testing

Phenotypic and genotypic susceptibility testing were performed as described previously [23]. Briefly, phenotypic antimicrobial resistance was assessed using the broth microdilution method with RAPMYCOI Sensititre™ titration plates, following CLSI standard M24 document [24]. The antibiotics tested included cotrimoxazole, ciprofloxacin, moxifloxacin, cefoxitin, amikacin, doxycycline, tigecycline, clarithromycin, linezolid, imipenem, and tobramycin. Genotypic susceptibility testing involved the GenoLyse® DNA extraction kit and GenoType® Mycobacterium NTM-DR test to identify MABS subspecies and resistance mutations in the *erm(41), rrl*, and *rrs* genes.

### Bacterial strains and growth conditions

Clinical and the laboratory ATCC 19977 reference [25] strains were grown in 10 mL of liquid Middlebrook 7H9 broth (Difco, BD Bioscience) in 25 mL untreated tissue culture flasks (Glass Chemicals, Spain), supplemented with 10% Middlebrook OADC (oleic acid-albumin-extrose-catalase; BBL, Becton-Dickinson), 0.2% glycerol, and 0.5% Tween 80 at 37°C with shaking at 200 rpm.

### Preparation of bacterial inoculum for subsequent infection of THP-1 cells

Bacterial inocula were prepared using two different methods. (i) Bacteria were initially collected at the logarithmic phase, centrifuged at 5000 ×  g for 10 minutes, and washed three times in phosphate-buffered saline (PBS). To prevent clumping, the liquid cultures were consistently passed 10 times through a syringe with a 25G-needle and vortexed with 4 mm glass beads for 10 seconds. The bacterial suspension was diluted in PBS to an OD of approximately 1, and the exact OD was measured at 600 nm using a BioPhotometer Plus® spectrophotometer (Eppendorf AG, Germany). Viable counts were obtained by plating serial dilutions on Columbia +  5% sheep blood agar plates (bioMérieux, France), followed by incubation at 37°C for one week. This procedure allowed us to calculate the CFU/OD ratio for each strain, however this procedure was not robust enough (see results section). (ii) Alternatively, aliquots of bacterial suspensions were stored at -80 ºC and used for further infections. Three aliquots of each strain were thawed one week after preparation, and the number of microorganisms was evaluated by plating 10-fold dilutions of the bacterial suspension. The remaining aliquots were kept frozen at − 80 °C until required. This procedure enabled us to determine the precise concentration (CFU/mL) for each frozen strain.

### Growth curves and doubling time calculations

To study the growth kinetics and doubling times of clinical isolates, we set up precultures from frozen stocks that, after 24 h, were reinoculated in fresh medium adjusting OD to 0.01 (time 0). Subsequently, OD was measured every 24-h for 5 days and viable counts were obtained by plating serial dilutions at 24 and 48 hours. The bacterial doubling time (BDT) was calculated using the following formula: BDT =  t *  ln(2)/ ln(N(final time)/ N(initial time)), where *N(final time)* is CFU/mL at 48 hours, *N(initial time)* is CFU/mL at 24 hours, and *t* is 24 hours *(final time* (48h) *– initial time* (24h)).

### Infection of THP-1 cells

THP-1 cells were cultured in 75 cm^2^ flasks in complete medium (CM): RPMI medium (Gibco™, Thermo Fisher Scientific, USA) supplemented with 10% heat-inactivated fetal bovine serum (FBS, Biowest, France), 100 U/mL penicillin, 100 µg/mL streptomycin and 50 µg/mL gentamycin (Gibco™, Thermo Fisher Scientific, USA) at 37 °C and 5% CO_2_. To differentiate THP-1 monocytes into macrophage-like cells, cells were treated with 50 ng/mL of phorbol 12-myristate 13-acetate (PMA), seeded at 10^5^ cells/well in 96-well plates (Nunc™ MicroWell™ 96-Well, Thermo Fisher Scientific, USA) and incubated for 72h. Following CM removal, the monolayers were washed with Dulbecco’s PBS (dPBS, Gibco™, Thermo Fisher Scientific, USA) and infected at a multiplicity of infection (MOI) of 1.

Bacterial inocula were prepared by first collecting cells at mid -logarithmic phase by centrifuging at 5000 ×  g for 10 minutes and washing three times with PBS. The OD was then adjusted in RPMI supplemented with 10% non-inactivated FBS and without antibiotics (incomplete medium, IM), to achieve a concentration of 10^6^ CFU/mL, based on the previously calculated CFU-OD ratio for each strain. Additionally, bacterial inocula were prepared by adding the necessary microliters from each previously frozen aliquot to IM, achieving a concentration of 10^6^ bacteria/mL. Thus, adding 100 µ L of these preparations provided 10^5^ bacteria per well. To confirm that the correct inoculum was added, all inocula used for infection experiments were plated and CFUs were counted. Then, infection was left to progress for 2 hours. Non-infected control macrophages received only IM, and all experiments were prepared in triplicate. Further, macrophages were washed three times in dPBS to remove extracellular bacteria and either treated or not with amikacin (250 µg/mL) for 1 h. Further, cells were washed again three times in dPBS and subsequently maintained in IM with or without 50 µg/mL amikacin for the duration of the experiment. To ensure consistency across both methodologies and maintain the same number of washes, cells were washed three times in dPBS, and the medium was replaced every 24 hours.

### Quantification of intracellular bacterial growth

To enumerate intracellular bacterial cells, infected THP-1 cells were washed with dPBS at each post-infection time to remove any residual amikacin (which could inhibit bacterial growth on solid media) and to eliminate any remaining extracellular bacteria and lysed in PBS containing 0.1% Triton X-100. After 5 minutes, bacterial suspensions and cell debris were serially diluted and plated on Columbia +  5% sheep blood agar plates.

### THP-1 viability measurements

For THP-1 viability experiments, infected monolayers were washed as above and treated with dPBS containing 2 mM Carboxyfluorescein diacetate succinimidyl ester (CFDA-SE Cell Proliferation Assay Kit, Bio-Rad) at room temperature (RT) for 15 minutes, washed three times with dPBS, and imaged using the EVOS FL (Thermo Fisher Scientific, Waltham, MA, USA) fluorescence microscope. Images were captured from three different fields of each well, and cell counting was performed using ImageJ software [26].

### Statistical analysis

Qualitative variables were presented with their frequency distributions (e.g., gender, MABS isolation). The association between qualitative variables (MABS isolation vs. sex) was evaluated using Fisher’s exact test because more than 20% of the expected values were less than 5.

Quantitative variables were reported with their mean value and standard deviation (SD) or Confidence Interval of 95% (CI 95%) when the data followed a normal distribution, and with median and interquartile range (IQR) when the data were not normally distributed. The Shapiro-Wilk test was used to assess whether the data followed a normal distribution, and the non-parametric Mann-Whitney U test was employed to compare age distributions between groups (with MABS isolates vs. without MABS isolates), due to the deviation from normality in the MABS isolation group. When data followed a normal distribution, t-tests were employed. Student´s-test for inequal variances (Welch’s test) was employed when Levene’s test indicated unequal variances, while Student’s t-test was used when variances were equal.

One-way ANOVA and two-way ANOVA, with adjustments for sphericity using the Geisser-Greenhouse correction, followed by Dunnett’s post hoc test, were used to analyse the results of the internalisation and THP-1 viability experiments.

A significance value of 5% was accepted for all tests. Statistical analyses were performed with Stata 17.0 and GraphPad Prism 8.3.

## Results

### Prevalence and clinical features of *M. abscessus* in the Spanish cohort

The study included 148 patients with CF. To date, 28 isolates of MABS have been found from 16 different patients, resulting in a prevalence of 10.8%. Among these patients, eight (5.4%) had one isolate, four (2.7%) had two isolates, and another four (2.7%) had three isolates. In this preliminary study, only the first isolate from each patient was stored for further analyses.

Among the 148 patients, 81 were men (54.7%). The mean age was 29.3 (SD =  13.9) and 29.6 (SD =  13.8) years for men and women, respectively. The median age of patients with MABS isolates was 24.4 years (IQR: 15.9-28.9) compared to 28.6 years (IQR: 20.8-38.3) for patients without MABS isolates, (p =  0.049). Regarding sex, 75.0% (12/16) of the patients with MABS isolation were male, compared to 52.3% (69/132) in the group without MABS isolation. The association between sex and MABS isolation was not statistically significant (p =  0.1) (Table 1).

**Table 1:** Age and sex distribution of CF patients by MABS culture results.

	MABS culture		
	Negative	Positive	p-value
Age Median (IQR)	28.6 (20.9-38.3)	24.4 (15.9-28.9)	0.049 ^a^
Male N (%)	69 (52.3%)	12 (75.0)	0.1^b^
Female N (%)	63 (47.7)	4 (25.0)	

^a^Mann-Whitney U test, exact p-value.

^b^Fisher’s exact test.

Out of 16 MABS clinical isolates, 13 (81.25%) were identified as MABSa and 3 (18.75%) as MABSm (strains 2, 8 and 16). No isolates of MABSb were found.

**Fig 1:**
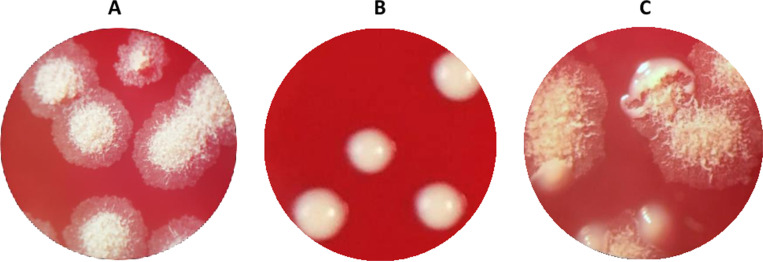
Example of morphotypes: A rough; B: smooth; C: mixed (strain 14).

Regarding morphology, eight strains were initially classified as R and another eight as S morphotypes. However, in subsequent experiments, a small proportion of R colonies was observed in strains 4 and 14, initially classified as S. Nevertheless, due to the predominance of S colonies during the experiments, these strains were analysed within the S group. Specifically, we found that 50% (8/16) were R, 37.5% (6/16) of the isolates were S, and 12.5% (2/16) exhibited a mixed morphology. Fig 1 shows an example of the phenotypic characteristics of colonies from each morphotype.

### Phenotypic and genotypic susceptibility testing

Table 2 summarises the distribution and interpretation of Minimum Inhibitory Concentrations (MICs) by subspecies. The highest *in vitro* resistance rates among MABS isolates were observed for doxycycline (100.0%), tobramycin (87.5%), ciprofloxacin (87.5%), moxifloxacin (75.0%), and cotrimoxazole (56.3%). In contrast, isolates demonstrated lower resistance rates to amikacin (18.8%), imipenem (12.5%), linezolid (6.3%), and cefoxitin (0.0%). Among MABSa isolates, 10 out of 13 exhibited inducible resistance to clarithromycin, while the remaining three, harbouring the T28C polymorphism in the *erm(41)* gene, remained susceptible after 14 days of incubation. Only one isolate (MABSm) demonstrated acquired resistance to clarithromycin, carrying a mutation in the *rrl* gene at position A2058G, as identified by the GenoType® test. Regarding amikacin resistance, 3 out of 16 isolates showed *in vitro* resistance: one belonged to the MABSm subspecies (strain number 16) and the other two to the MABSa subspecies (strains numbers 9 and 13). The GenoType® test detected mutations in the *rrs* gene in all these resistant strains. There was a 100% concordance between the GenoType® test results and phenotypic susceptibility testing across all studied genes (*erm(41), rrl,* and *rrs*), with all 16 isolates showing consistent results.

**Table 2:** Antimicrobial susceptibility testing of MABS isolates.

Drug Subsp.	Number of strains at each MIC (µg/mL)												N(%) Resistant
	≤0.06	0.12	0.25	0.5	1	2	4	8	16	32	64	128	
Cotrimoxazole													
MABSa (n=13)			1		1	2			9^↑^				9 (69.2)
MABSm (n=3)						3							0 (0.0)
Ciprofloxacin													
MABSa						1^*^	4	8^↑^					12 (92.3)
MABSm				1				2^↑^					2 (66.7)
Moxifloxacin													
MABSa					1	2^*^	2		8^↑^				10 (76.9)
MABSm					1		2						2 (66.7)
Cefoxitin													
MABSa								1	8	4^*^			0 (0.0)
MABSm									2	1^*^			0 (0.0)
Tigecycline ^¥^													
MABSa	*2*	*3*	*6*	*2*									¥
MABSm	*1*	*2*											¥
Amikacin													
MABSa							3	4	4			2^↑^^,^^a^	2 (15.4)
MABSm							2					1^↑^^,^^a^	1 (33.3)
Drug Subsp.	Number of strains at each MIC (µg/ml)												
	≤0.06	0.12	0.25	0.5	1	2	4	8	16	32	64	128	
Clarithromycin (3 days)													
MABSa	5		2	4	2								0 (0.0)
MABSm	2									1^↑^^,^^b^			1 (33.0)
Clarithromycin (14 days)													
MABSa		2^c^			1^c^					10^↑^			10 (76.9)
MABSm^d^	1	1											0 (0.0)
Linezolid													
MABSa					2	2	1		7^*^		1^↑^		1 (7.7)
MABSm					1		2						0 (0.0)
Imipenem													
MABSa							4	3^*^	5^*^	1			1 (7.7)
MABSm								2^*^				1^↑^	1 (33.3)
Tobramycin													
MABSa						1	1^*^	3	7	1^↑^			11 (84.6)
xMABSm								1	2				3 (100.0)

Strains with MICs interpreted as **Resistant** are highlighted in **bold**.

* Indicates strains with MICs interpreted as Intermediate.

¥ No established breakpoints exist for tigecycline; the number of strains at each MIC is shown in *italic* type.

↑ Off-scale high MICs were included at the next highest concentration.

^a^Strains harbouring a *rrs* mutation.

^b^MABSm strain harbouring a *rrl* mutation (acquired resistance to clarithromycin).

^c^Strains harbouring the T28C mutation at *erm(41),* not showing inducible resistance*.*

^d^MABSm strain showing acquired resistance to clarithromycin was excluded from this analysis.

### Growth curves measured by OD, determination of CFU/OD and CFU/mL relationship, and doubling time

**Fig 2:**
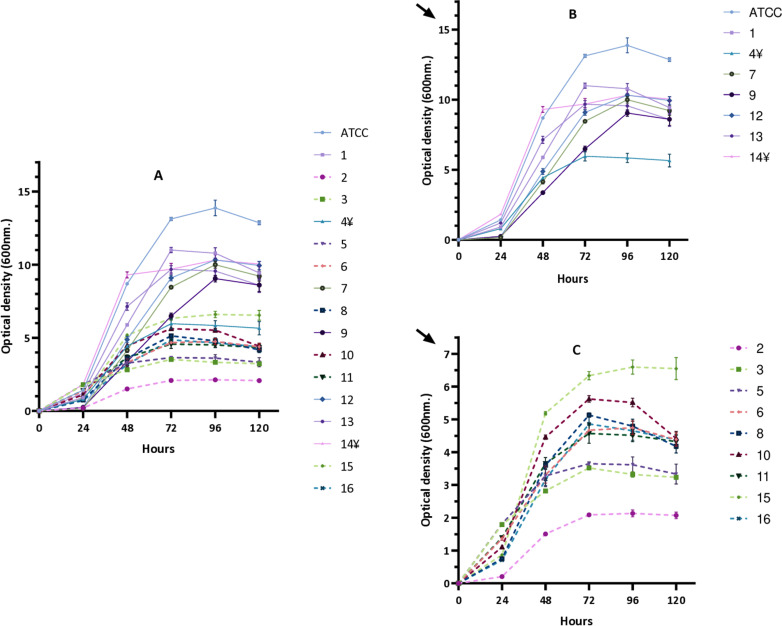
Growth dynamics of smooth and rough strains over time by measuring OD. **Panel A** represents data for all strains**. B:** smooth strains**; C:** rough strains with rescaled OD values (see arrows). Although smooth (**B**) and rough (**C**) strains manifested similar growth dynamics, smooth strains reached higher OD values. Data are mean and SD from three independent experiments.

We initially monitored the growth by measuring OD and found differences between isolates displaying different morphotypes (Fig 2, Table 3). We noted that strains from both morphotypes followed a similar growth dynamic. However, S strains reached higher OD values compared to R strains. Specifically, no significant differences in OD were observed between the morphotypes at 24 h. Moreover, S strains consistently exhibited significantly higher OD values compared to R strains at 48, 72, 96, and 120 hours. In both morphotypes, the mid-logarithmic phase was registered between 24 and 48 hours.

**Table 3:** Optical density results at each time point, compared by morphology.

S strains				R strains			Difference in means
Hours	Mean-OD	SD ^a^	CI 95%	Mean-OD	SD ^a^	CI 95%	
24	0.85	0.61	0.34-1.36	1.11	0.53	0.70-1.52	-0.26^ns^
48	5.98	2.19	4.15-7.81	3.44	1.02	2.65-4.23	2.54^*^
72	9.19	2.32	7.25-11.14	4.49	1.26	3.53-5.46	4.70^**^
96	9.97	2.21	8.11-11.82	4.43	1.29	3.43-5.42	5.53^**^
120	9.30	2.00	7.63-10.97	4.10	1.21	3.16-5.03	5.20^**^

Student’s t-test.

* p <  0.05.

** p < 0.001.

^a^Standard deviation

Aiming at establishing an OD-CFU ratio we plated cultures from each strain at different time points and found that this approach does not provide consistent results being not reliable for further studies. Specifically, we adjusted 24 and 48-h cultures to an OD = 1 (CFU-OD = 1) performed serial dilutions and plated them in solid medium. We observed that the CFU-OD = 1 ratio was significantly higher at 48 hours compared to 24 hours for both morphotypes. While the CFU-OD = 1 ratio is similar between morphotypes at 24 hours, it increases approximately 10-fold for the R strains at 48 hours (Table 4). Importantly, when we performed this procedure to infect the cells, we found that this ratio was not robust enough to accurately adjust the initial bacterial inoculum to 10⁶ bacteria/mL, resulting these inocula in a median of 7.2 ×  10⁵ CFU/mL (range: 1.1 ×  10⁴ – 1.2 ×  10^7^). To circumvent this, we evaluated whether freezing aliquots of these bacterial suspensions at -80°C could be a reliable method to calculate the CFU/mL ratio. This approach proved to be much accurate in achieving a known bacterial inoculum concentration for subsequent THP-1 infections, with a median of 9.9 ×  10⁵ CFU/mL (range: 7.5 ×  10⁵ – 1.3 ×  10⁶). Therefore, all subsequent experiments requiring a known bacterial inoculum such as THP-1 infection and viability assays, were performed using this CFU/mL procedure, which involved freezing aliquots.

**Table 4:** CFU-OD = 1 ratio at 24 and 48 hpi, compared by morphology.

	S strains		R strains			
Hours	CFU-OD = 1	SD	CFU-OD = 1	SD	p-value ^a^	Prob.(R > S) ^b^
24	1.18e + 09	5.64e + 08	1.63e + 09	1.56e + 09	0.70	0.56
48	3.52e + 09	2.33e + 09	1.11e + 10	1.24e + 10	**0.02**	0.83
p-value-(column)^a^	**0.03**		**<0.001**			
P(48h > 24h)^b^	0.83		0.95			

^a^Mann-Whitney U test, exact p-value;

^b^Probability of superiority.

Regarding the bacterial doubling time, calculated using the formula “BDT = t * ln(2)/ ln(N(final time)/ N(initial time))”, no significant differences were observed between S strains (5.3 hours, 95% CI: 4.7-5.9) and R strains (5.5 hours, 95% CI: 5.0-6.0), p = 0.6. (S1 Table).

### Quantification of intracellular MABS survival in THP-1 cells

**Fig 3:**
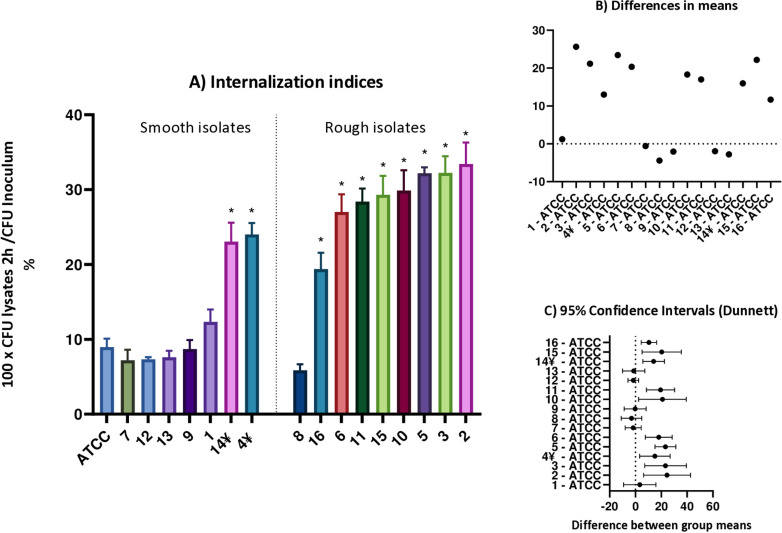
Internalisation indices for each MABS strain, compared with the ATCC strain. A: Error bars indicate the SD, based on the results from three independent experiments. *  p < 0.05. One-way ANOVA with Dunnett’s post hoc test. B: Differences in means for internalisation indices, comparing each strain to the ATCC strain. C: 95% confidence intervals for the differences in group means, using Dunnett’s method for multiple comparisons. ¥ Strains exhibiting mixed morphology.

Using THP-1 cells as a model, we initially investigated the internalisation indices for each strain by counting the internalised bacterial cells 2 h after infection. A significantly higher internalisation index mean was observed for the R morphotypes (26.4% ±  7.1%) compared to the S morphotypes (12.4% ±  8.8%), p = 0.003 (Fig 3). We noted that strains 4 and 14, which manifested a mixed morphology, showed internalisation indices similar to that of R strains. Conversely, R strain 8 exhibited a notably low internalisation index compared to other R strains.

**Fig 4:**
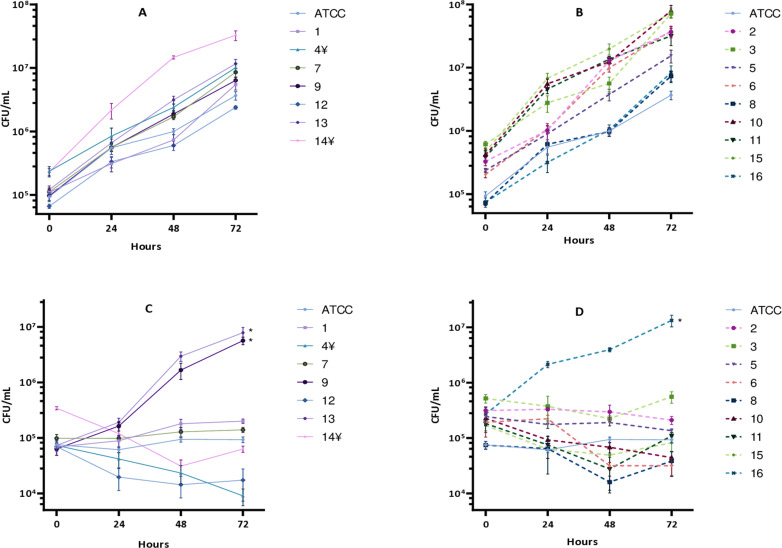
Intracellular growth of smooth and rough strains under amikacin or amikacin-free conditions. A, B: Intracellular growth of smooth and rough strains, respectively, in the absence of amikacin. C, D: Intracellular growth of smooth and rough strains, respectively, in the presence of amikacin. Error bars indicate the standard deviation, based on the results from three independent experiments. *  Amikacin-resistant strains. ¥ Strains exhibiting mixed morphology.

Subsequently, we explored the intracellular survival of MABS isolates in a time course experiment. We found that throughout the infection experiments R strains exhibited higher mean bacterial numbers compared to S strains at all time points and conditions evaluated. Importantly, amikacin is typically used in these experiments to prevent the proliferation of extracellular bacteria, and it has been shown that does not penetrate inside THP-1 cells [27]. In this context, amikacin-resistant strains (strains 9, 13 and 16) were excluded when this antibiotic was used during experimental infections, except at 2 hours post-infection (hpi). When cells were maintained in medium with amikacin throughout the infection experiment, significant differences were observed in the mean number of intracellular MABS between S and R strains at 2 hpi, with R strains showing higher cell counts (2.4x10^5^ ±  1.2 × 10^5^ CFU/mL) compared to S strains (1.1x10^5^ ±  9.6x10^4^ CFU/mL), p = 0.02. However, these differences disappeared at 24, 48 and 72 hpi (p = 0.05; p = 0.5; p = 0.4, respectively). These analyses compare the mean values of S and R strains, based on individual data shown in Fig 4C and 4D. Conversely, when amikacin was not included in the medium after infection, significant differences were observed at 2 hpi, with R strains showing higher cell counts (3.1 × 10^5^ ±  1.8 × 10^5^CFU/mL) compared to S strains (1.3x10^5^ ±  6.4x10^4^ CFU/mL), p =  0.02. Similarly, R strains also exhibited higher CFU/mL means compared to S strains at 24 and 48 hpi, but these differences were not statistically significant (p = 0.05 and p = 0.06, respectively). However, bacterial loads were significantly higher in R strains (4.0 × 10^7^ ±  9.4 × 10^6^ CFU/mL) at 72 hpi relative to S strains (1.0 × 10^7^ ±  3.4 × 10^6^ CFU/mL), p =  0.01 (Fig 4A and 4B).

### Viability of MABS-infected THP-1 cells

**Fig 5:**
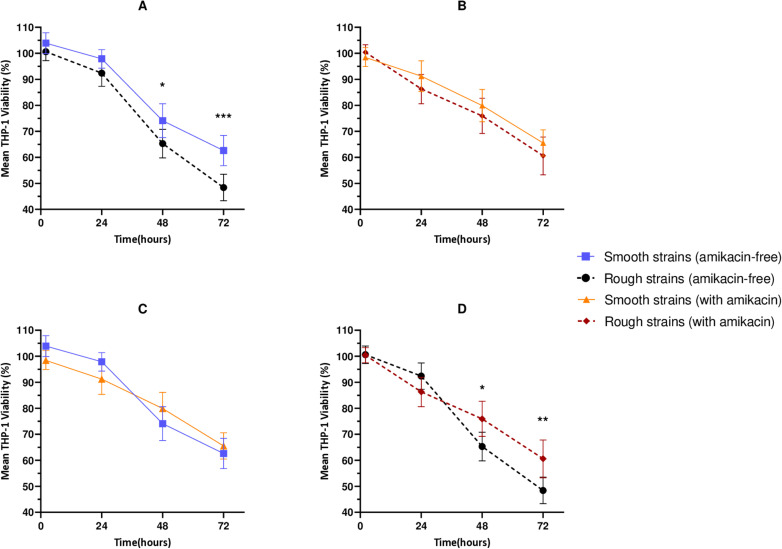
Effect of infection conditions on THP-1 cell viability. Data represent the mean ±  95% confidence interval of three independent experiments. Panels **A, B**: comparison of the viability of THP-1 cells infected with rough and smooth strains in amikacin-free medium (**A)** or amikacin-containing medium (B). Panels **C, D**: comparison of THP-1 cell viability in the absence or presence of amikacin for infections with **S (C)** and **R (D)** strains. Statistical significance was determined using a two-tailed Student’s t-test. *  **p** <  0.05; p < 0.01; ***p <  0.001.

We next studied the viability of THP-1 cells after infection using a fluorescence dye. We found no significant differences in THP-1 cell viability between cells infected with S or R strains in the absence of amikacin at 2 and 24 hpi (Fig 5A, section “a” in S2 Table and S1B and S1C Fig. These differences were statistically significant at 48 and 72 hpi where R strains showed an enhanced capacity to reduce THP-1 viability relative to macrophages infected with S strains (Fig 5A, section “a” in S2 Table and S1B and S1C Fig. Conversely, we found no significant differences in cell viability between cells infected with S or R strains at any time point when amikacin was used (Fig 5B, section “b” in S2 Table and S1E and S1F Fig. More detailed information on the statistical analyses is provided in S2 Table.

**Fig 6:**
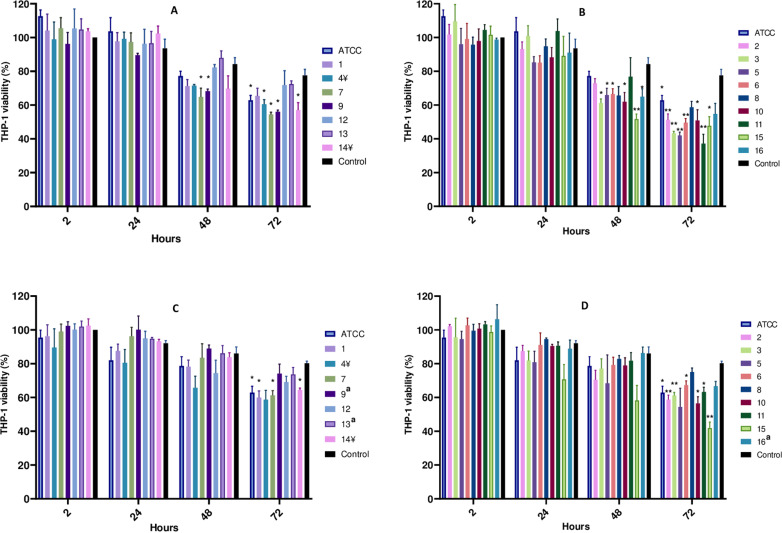
Impact of MABS strains on THP-1 cell viability under untreated and amikacin-treated conditions. Viability percentages are shown relative to the uninfected control at 2 hpi, allowing for the observation of natural viability loss in uninfected THP-1 cells up to 72 hours. Statistical analyses compared the viability of THP-1 cells infected with each strain to the corresponding control at each time point. A, B: Viability of THP-1 cells infected with smooth and rough strains, respectively, in the absence of amikacin. C, D: Viability of THP-1 cells infected with smooth and rough strains, respectively, in the presence of amikacin. Error bars indicate the SD, based on the results from three independent experiments. Two-way ANOVA with Dunnett’s post hoc test. *  p < 0.05, ** p < 0.01. ^**a**^ Amikacin-resistant strains (numbers 9, 13 and 16) were excluded from this statistical analysis. ¥ Strains exhibiting mixed morphology.

When analysing the effect of individual strains on THP-1 cell viability (Fig 6), the two-way repeated measures ANOVA test revealed significant impacts from both the time and the strain factor. In experiments conducted without amikacin (Fig 6A and 6B and S1A–S1C Fig, significant differences in THP-1 cell viability compared to uninfected controls were detected at 48 hpi, with further significant disparities noted at the final time point, and with R strains demonstrating more pronounced effects. In contrast, in experiments with amikacin (Fig 6C and 6D and S1E and S1F Fig, significant differences in THP-1 cell viability were first observed at the final time point. Viability data, together with intracellular CFU counts, are presented in S2 Fig, illustrating potential relationships between these parameters across different strains and time points.

**Fig 7:**
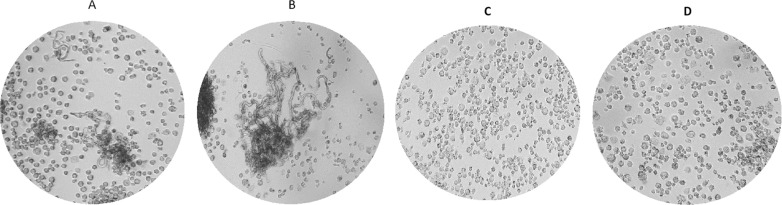
Example of cord-like aggregates produced by the rough strain number 15 at 48 hpi (A) and 72 hpi (B) in the experiment without amikacin. These cords were not observed in smooth strains at 48 hpi (C) or 72 hpi (D).

Notably, we could observe multiple cord-like aggregates (Fig 7A and 7B) for most R strains despite numerous washes between time points and before staining the cells when amikacin was not included in the experiment. Quantitative assessment of extracellular CFUs under these conditions was not feasible due to the experimental limitations; however, the formation of these prominent aggregates provides qualitative evidence of significant extracellular growth.

Finally, when comparing the effects of amikacin-containing medium versus amikacin-free medium on THP-1 cell viability, no significant differences were observed when THP-1 cells were infected with S at any time point. In contrast, for R strains, significant differences were observed at 48 and 72 hpi. Notably, we measured a mean viability of 65.3% (95% CI: 59.8 - 70.8,) without amikacin (section “a” in S2 Table, in bold) and 75.9% (95% CI: 69.2 - 82.6) with amikacin (section “b” in S2 Table, in bold) at 48 h, p = 0.01; and a mean viability of 48.4% (95% CI: 43.3 - 53.5) without amikacin (section “a” in S2 Table, in bold and italics) and 60.5% (95% CI: 53.3 - 67.8) with amikacin (section “b” in S2 Table, in bold and italics) at 72 h, p = 0.006.

## Discussion

The objective of this study was to characterise the growth dynamics and virulence of *M. abscessus* clinical strains, with a particular focus on those isolated from CF patients. We aimed to compare the infection kinetics and proliferation capacities of S and R *M. abscessus* strains within THP-1 cells under various experimental conditions. Additionally, we sought to optimise and identify the limitations of the methodologies employed, including the assessment of the impact of amikacin on infection experiments.

We found that nearly 11% of patients had at least one MABS isolate during the study period. Regarding demographic data, the group with MABS isolates was younger than the group without. The difference was marginally significant (p = 0.049), but it could suggest that younger individuals might be more susceptible to MABS or more frequently exposed in our setting. In the literature, it is commonly described that CF patients with MABS isolates are younger than those with isolates of other NTM, but it is not usual for them to be younger than groups without NTM isolation [5,28,29]. As for sex, 75.0% (12/16) of the patients with MABS isolation were male. Although the association between sex and MABS isolation was not statistically significant, the higher percentage of male patients with MABS isolation in our study is noteworthy. Nonetheless, in other CF epidemiology studies, either no sex-related differences have been found [5] or, in some cases, a higher prevalence in females has been reported [30]. However, few studies specifically examine the epidemiology of MABS in CF patients, and most of them focus on NTM infections in the general population or a mix of patients with various diseases other than CF [31,32]. Therefore, this observed trend favouring females in NTM infections may not necessarily apply to the CF patient population. These discrepancies call for further investigation to understand the potential role of sex and age in MABS infection dynamics and to clarify these findings.

We observed that 50% of the isolates were R, 37.5% were S, and 12.5% exhibited a mixed morphology, which is consistent with findings from a similar study conducted in Germany [33]. MABSa accounted for most isolates in our study (81.3%), with no MABSb strains identified. This agrees with global data, where MABSa is commonly reported as the most prevalent subspecies, followed by MABSm, while MABSb is infrequently found. Nonetheless, our findings show a higher proportion of MABSa than that of typically reported [34].

Regarding susceptibility testing, we observed high *in vitro* resistance rates (>85%) to doxycycline, tobramycin, ciprofloxacin and moxifloxacin (75%) consistent with findings from previous studies [23,35,36]. Cotrimoxazole also displayed high resistance rates, however, compared to an epidemiological study from our region [23], the present study showed notably lower resistance rates to cotrimoxazole (56.3% vs. 82.3%). Conversely, we identified a considerably higher resistance rate to amikacin (18.8% vs. 2.1%), a finding that also contrasts with most studies where amikacin resistance rates typically remain around 5% [36–39]. These differences may be attributed to the fact that the present study exclusively focused on CF patients, who have different host characteristics and are typically more frequently exposed to different antimicrobial treatments. The 68.8% of strains exhibited either inducible (10 MABSa) or acquired (1 MABSm) macrolide resistance. This high percentage of resistance is attributed to the large proportion of MABSa isolates, which are known to commonly express inducible resistance to macrolides due to the presence of the *erm*(41) gene [8]. These results are particularly concerning since clarithromycin and amikacin are key drugs used in the treatment of MABS infections [12]. We found the lowest resistance rates to imipenem (12.5%), linezolid (6.3%) and cefoxitin (0%), consistent with previous results in our region, although imipenem resistance was significantly lower than typically reported [36–38]. Finally, tigecycline has proven effective *in vitro* against RG-NTM and is currently recommended for treating MABS infections according to existing guidelines (11, 38). Despite the absence of an established susceptibility breakpoint, tigecycline showed strong *in vitro* activity against all isolates, with MICs ≤  0.5 μg/mL.

Next, we studied growth kinetics and found that S or R strains followed a similar pattern. However, S strains reached significantly higher maximum OD values compared to R strains, especially at later time points. This discrepancy could be due to more pronounced aggregation phenomena in R strains, particularly at later time points. Notably, unreliable OD results have also been described in other studies involving different mycobacterial species [40,41] including *Mycobacterium tuberculosis*, also recognised for its tendency to form aggregates [42]. Despite efforts to disrupt aggregates by shaking with glass beads and syringe passages, R strain cultures remained less homogeneous. The bacterial aggregation led to an underestimation of OD measurements, making the CFU-OD = 1 ratio unreliable for establishing a known bacterial inoculum, especially for R strains. The doubling time observed for S (5.3 hours) and R strains (5.5 hours) were nearly identical, and very similar to those reported in a previous study utilising S and R laboratory strains [43]. These similarities reinforce the conclusion that the elevated CFU-OD = 1 ratio observed in R strains is attributable to aggregation effects rather than inherent differences in growth rates. After using this method and recounting the CFUs to verify whether the inocula contained the necessary bacterial concentration, it was observed that the concentration ranges obtained were highly variable (median = 7.2 ×  10⁵ CFU/mL; range: 1.1 ×  10⁴ – 1.2 ×  10^7^). This suggests that procedures such as centrifugation and washing of the broth culture during the logarithmic phase might not be robust enough to maintain a consistent OD-CFU ratio across experiments. Therefore, to ensure accurate and consistent infection inocula, we performed a procedure involving the freezing of aliquots of known concentrations, as performed in previous studies [27,44]. This approach provided robust and reproducible data (median = 9.9 ×  10⁵ CFU/mL, range: 7.5 ×  10⁵ – 1.3 ×  10⁶), accommodating the unique characteristics and growth phase variations of each strain. Consequently, we selected this method for preparing bacterial inocula for subsequent infection experiments.

When examining intracellular growth by using the THP-1 cell infection model we observed that R strains reached higher CFU/mL compared to S strains at all time points and medium conditions. However, statistically significant differences were observed only at specific time points. In the absence of amikacin, significant differences between R and S strains were observed at both 2 hpi and 72 hpi. A recent study also reported that the proportion of infected macrophages was significantly higher among clinical R strains compared to S strains at 72 hours, though no differences were observed at 4 hpi [45]. Although differences at 24 and 48 hpi were not statistically significant, they were remarkably close to significance. The significant differences observed at 72 hpi could be due to the aggregation and clumping behaviours characteristic of R strains, which formed extracellular cord-like aggregates and persisted adhered to the wells despite several dPBS washes (Fig 7). Additionally, this fact might allow for the reinternalisation of the mycobacteria, which could more significantly affect macrophage viability in the absence of amikacin, as observed in the viability experiments.

Notably, when using amikacin after infection, significant differences in CFU numbers were only noted at 2 hpi, where R strains showed higher numbers. This suggests that the R morphotype might be more efficiently taken up by macrophages and/or phagocytised in small bacterial aggregates, unlike S strains, which are frequently phagocytised individually, as described previously [43,46]. The observed differences in internalisation indices imply that certain strains have a greater capacity to invade host cells compared to the ATCC reference strain. These variations may impact the capacity of macrophages to eliminate intracellular bacteria. The significantly higher internalisation indices observed in some strains (specially R morphotype) underscore their potential for enhanced pathogenicity, highlighting the need for further investigation into the underlying mechanisms. However, as previously mentioned, some strains deviated from this trend. Strains 4 and 14 displayed a mixed morphology and, despite being predominantly smooth, their internalisation indices were closer to that of rough strains. In contrast, rough strain 8 exhibited a notably low internalisation index. These inconsistencies encourage further investigation to understand why its behaviour differs from that of the other rough strains. In the literature, different internalisation indices have been described depending on experimental conditions such as the duration of cell exposure to infection, the multiplicity of infection or the cells used [46,47], but when comparing S and R internalisation rates, this index is generally higher for R strains [46].

In the THP-1 viability assays, significant differences in THP-1 cell viability were observed when comparing infections with R versus S strains in the amikacin-free experiment at 48 and 72 hpi (Fig 5A and section “a” in S2 Table). Conversely, no statistically significant differences in mean viability were measured in the presence of amikacin (Fig 5B and section “b” in S2 Table). Despite this, several R strains exhibited a more pronounced reduction in THP-1 cell viability at 48 and 72 hpi (without amikacin, Fig 6A and 6B) or at 72h (with amikacin, Fig 6C and 6D), when compared individually to uninfected THP-1 controls. These findings are consistent with previous studies, which also reported that R strains caused higher macrophage death at later time points compared to S strains [46,48]. However, Aulicinio et al. found a strong similarity in transcriptional responses to both morphotypes, while noting a distinct inflammatory response. Specifically, R strains triggered a less pronounced inflammatory reaction, which may facilitate their persistence within macrophages [48].

Finally, when comparing THP-1 cell viability under conditions with and without amikacin within the same morphotype, no significant differences were observed in the impact on THP-1 cell viability when infected with S strains at any time point analysed (Fig 5C), indicating that the presence or absence of amikacin had little effect on viability results in this morphotype. In contrast, R strains showed a significantly greater impact on THP-1 viability at 48 and 72 hpi when amikacin was absent. Specifically, THP-1 viability was reduced by 10.6% (p = 0.01) at 48 hpi (Fig 5D and S2 Table, in bold) and by 12.2% (p = 0.006) at 72 hpi in the absence of amikacin (Fig 5D and S2 Table, in bold and italics). Overall, the R strains exhibited more significant impact in cell viability, especially in the experiments without amikacin and becomes more evident over time (Figs 5 and 6).

In the context of human infection, these findings highlight the complex dynamics of MABS infections, especially in CF patients. The higher CFU counts and the more significant impact on cell viability observed in the R strains suggest that this morphotype might have an enhanced ability to persist in the host. In real-world CF patients, these more aggressive behaviours of strains with R morphotype could translate into chronic and more difficult-to-treat infections [49]. The tendency of R strains to form extracellular cord-like aggregates may enhance its persistence in the host, evading both the immune response and antibiotic treatment.

However, the results reported herein should be considered in the light of some limitations. This study was conducted in an *in vitro* model, which does not fully replicate the complexity of human infections. In real patients, numerous factors, such as the overall host’s immune response, co-existing pathogens, and the unique environment of the CF lung, influence the course of infection. Therefore, while our results offer valuable insights into potential mechanisms of persistence and resistance in MABS infections, further research is needed to determine how these findings translate to clinical settings and to develop more effective therapeutic strategies for managing these difficult-to-treat infections. Moreover, our findings highlight the limitations of commonly employed cellular infection assays. Using amikacin media to control extracellular growth ensures that colony quantification post-lysis reflects the intracellular environment. However, this approach underestimates the true infection burden by not taking into consideration the aggregation and extracellular fate of R strains and their enhanced tendency to lyse macrophages and exit into the extracellular medium. On the other hand, omitting amikacin allows for a more comprehensive understanding of bacterial dynamics, including escape and reinternalisation events, but can lead to overgrowth and complicate interpretation due to extracellular aggregates in this *in vitro* infection model. Finally, while this study included 148 patients, the number of MABS isolates was relatively low. This limited sample size may reduce the power to detect significant associations and restricts the generalisability of the findings to other CF populations. Nonetheless, few studies have explored this topic in depth, and published research in the field typically relies on only a few laboratory strains. Therefore, despite the sample size limitation, our study contributes important data to an underexplored area and underscores the need for continued investigation.

In summary, our study highlights the significant prevalence of MABS isolation among CF patients, particularly among younger individuals. The male predominance in MABS cases, although not statistically significant, prompts further epidemiological investigation. We found high resistance rates to several commonly used antibiotics, with both phenotypic and genotypic testing confirming these resistance patterns. Rough MABS strains showed increased aggregation, complicating OD-based quantification, especially over extended periods; therefore, we recommend preparing frozen aliquots of known concentration to ensure the robustness of such experiments. Most rough strains demonstrated enhanced pathogenicity compared to smooth strains, as evidenced by higher intracellular CFU counts and greater impact on THP-1 cell viability, especially in the absence of amikacin. The differences observed between both methodologies underscore the importance of evaluating results while considering the limitations of each. These observations suggest that rough strains may pose a greater threat to patients, highlighting the need for further investigation into the underlying mechanisms and emphasizing the importance of considering these differences in clinical management. It is also important to note that the genomic characterization of the strains and patient treatment outcomes are still being collected and will be reported once all patient visits have concluded.

## Supporting information

S1 Fig Fluorescence microscopy images at each time point. Representative fluorescence microscopy images of viability of uninfected cells (A, D), cells infected with a rough strain (B, E), and cells infected with a smooth strain (C, F) at each studied time point. Panels A-C show cells non treated with amikacin, while panels D-F show cells treated with amikacin.
(DOCX)

S2 Fig Relationship between intracellular Mycobacterium abscessus growth and THP-1 cell viability over time under amikacin or amikacin-free conditions. This figure displays the intracellular bacterial burden of Mycobacterium abscessus in THP-1 cells, measured as CFU/mL, along with the corresponding THP-1 cell viability over time. The left axis and data points represent the mean CFU/mL (log scale) of triplicates. The right axis and bars represent the percentage of THP-1 cell viability relative to the 2h non-infected control at each time point post-infection, expressed as the mean ±  standard deviation of triplicates. **A, B:** data for smooth and rough strains, respectively, in the absence of amikacin. **C, D**: data for smooth and rough strains, respectively, in the presence of amikacin. ¥ Strains exhibiting mixed morphology.
(DOCX)

S1 Table Mean bacterial doubling time by strain and by morphology. (DOCX)

S2 Table Viability of THP-1 cells infected with S and R strains under amikacin and amikacin-free conditions. (DOCX)
