# Supplementary material for: Infection model of THP-1 cells, growth dynamics, and antimicrobial susceptibility of clinical Mycobacterium abscessus isolates from cystic fibrosis patients: Results from a multicentre study
Source: PLoS One. 2025 Mar 31;20(3):e0319710. doi: 10.1371/journal.pone.0319710 (PMC11957364; doi:10.1371/journal.pone.0319710)
Supplement: S1 Fig — Representative fluorescence microscopy images of viability of uninfected cells (A, D), cells infected with a rough strain (B, E), and cells infected with a smooth strain (C, F) at each studied time point. Panels A-C show cells non treated with amikacin, while panels D-F show cells treated with amikacin. (DOCX) [file pone.0319710.s001.docx]

**
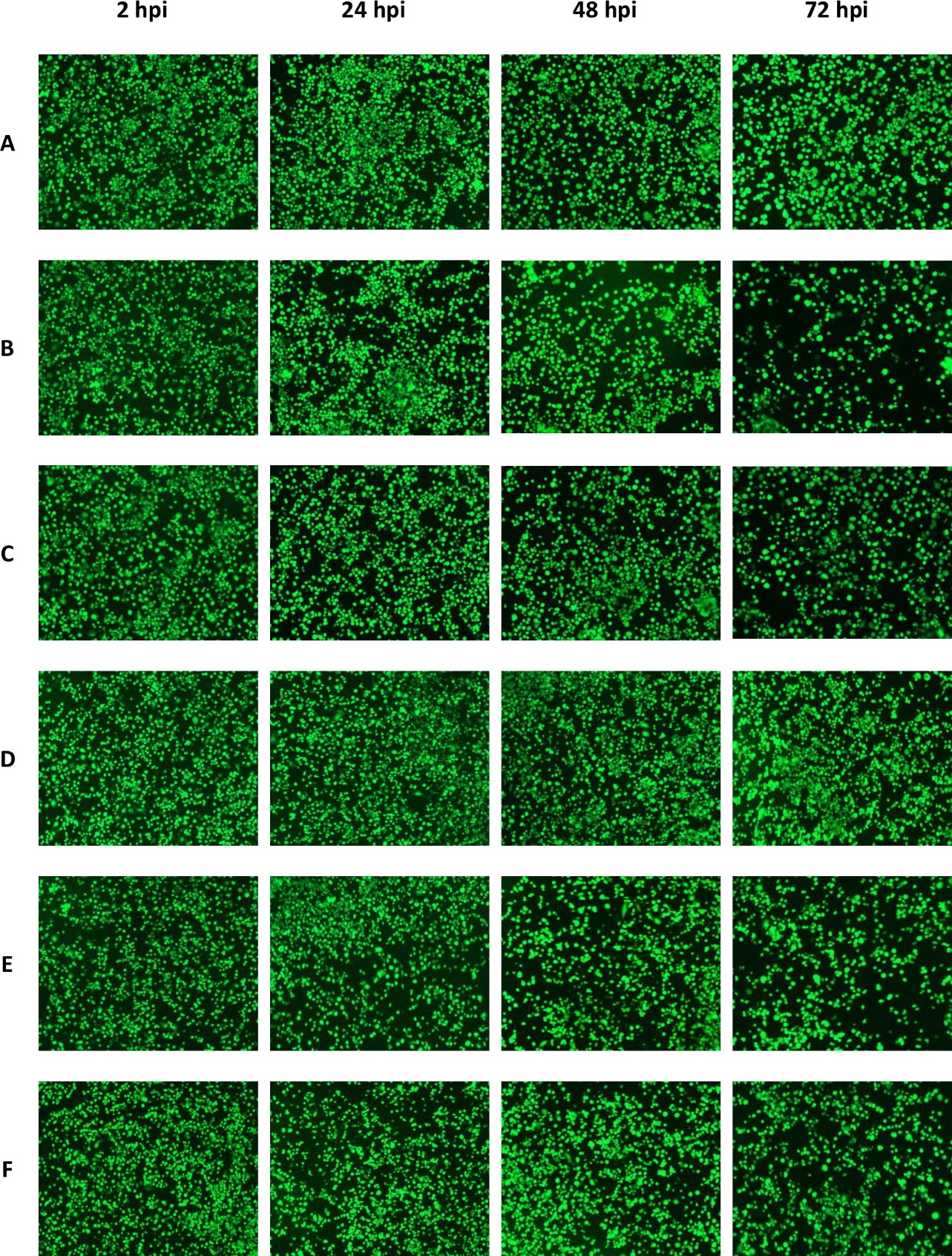
**

**S1 Fig. Fluorescence microscopy images at each time point.** Representative fluorescence microscopy images of viability of uninfected cells **(A, D)**, cells infected with a rough strain **(B, E)**, and cells infected with a smooth strain **(C, F)** at each studied time point. Panels A-C show cells non treated with amikacin, while panels D-F show cells treated with amikacin.
