# Supplementary material for: Infection model of THP-1 cells, growth dynamics, and antimicrobial susceptibility of clinical Mycobacterium abscessus isolates from cystic fibrosis patients: Results from a multicentre study
Source: PLoS One. 2025 Mar 31;20(3):e0319710. doi: 10.1371/journal.pone.0319710 (PMC11957364; doi:10.1371/journal.pone.0319710)
Supplement: S2 Fig — This figure displays the intracellular bacterial burden of Mycobacterium abscessus in THP-1 cells, measured as CFU/mL, along with the corresponding THP-1 cell viability over time. The left axis and data points represent the mean CFU/mL (log scale) of triplicates. The right axis and bars represent the percentage of THP-1 cell viability relative to the 2h non-infected control at each time point post-infection, expressed as the mean ± standard deviation of triplicates. A, B: data for smooth and rough strains, respectively, in the absence of amikacin. C, D: data for smooth and rough strains, respectively, in the presence of amikacin. ¥ Strains exhibiting mixed morphology. (DOCX) [file pone.0319710.s002.docx]

Left Y axis, plots, CFU/mL: Right Y axis, bars, THP-1 viability (%):

**S2 Fig. Relationship between intracellular *Mycobacterium abscessus* growth and THP-1 cell viability over time** **under amikacin or amikacin-free conditions.** This figure displays the intracellular bacterial burden of *Mycobacterium abscessus* in THP-1 cells, measured as CFU/mL, along with the corresponding THP-1 cell viability over time. The left axis and data points represent the mean CFU/mL (log scale) of triplicates. The right axis and bars represent the percentage of THP-1 cell viability relative to the 2h non-infected control at each time point post-infection, expressed as the mean ± standard deviation of triplicates. **A, B:** data for smooth and rough strains, respectively, in the absence of amikacin. **C, D**: data for smooth and rough strains, respectively, in the presence of amikacin. ¥ Strains exhibiting mixed morphology.
