# Supplementary material for: Infection model of THP-1 cells, growth dynamics, and antimicrobial susceptibility of clinical Mycobacterium abscessus isolates from cystic fibrosis patients: Results from a multicentre study
Source: PLoS One. 2025 Mar 31;20(3):e0319710. doi: 10.1371/journal.pone.0319710 (PMC11957364; doi:10.1371/journal.pone.0319710)
Supplement: S1 Table — (DOCX) [file pone.0319710.s003.docx]

| **S1 Table. Mean bacterial doubling time by strain and by morphology.** | | | | | | |
| --- | --- | --- | --- | --- | --- | --- |
| **S strains** | | | **R strains** | | |  |
| **Strain** | **Mean-BDT (hours)** | **SD^a^-BDT** | **Strain** | **Mean-BDT (hours)** | **SD^a^-BDT** |  |
| ATCC | 6.3 | 1.0 | 2 | 4.8 | 0.5 |  |
| 1 | 5.6 | 0.6 | 3 | 6.3 | 0.4 |  |
| 4 | 5.7 | 0.2 | 5 | 6.0 | 2.6 |  |
| 7 | 4.5 | 0.1 | 6 | 5.6 | 0.7 |  |
| 9 | 4.6 | 0.6 | 8 | 6.1 | 0.8 |  |
| 12 | 4.3 | 0.2 | 10 | 4.2 | 0.1 |  |
| 13 | 6.1 | 0.7 | 11 | 5.4 | 0.7 |  |
| 14 | 5.3 | 0.3 | 15 | 5.4 | 2.1 |  |
|  |  |  | 16 | 5.7 | 0.1 |  |
| **Total-S** | **Mean-BDT** | **CI 95%** | **Total R** | **Mean-BDT** | **CI 95%** | **p-value** |
|  | 5.3 | 4.7-5.9 |  | 5.5 | 5.0-6.0 | 0.6* |
| * Student´s T-test. ^a^ Standard deviation, based on the results from three independent experiments. **BDT**: bacterial doubling time. **S**: smooth; **R**: rough. | | | | | | |
