# Supplementary material for: Infection model of THP-1 cells, growth dynamics, and antimicrobial susceptibility of clinical Mycobacterium abscessus isolates from cystic fibrosis patients: Results from a multicentre study
Source: PLoS One. 2025 Mar 31;20(3):e0319710. doi: 10.1371/journal.pone.0319710 (PMC11957364; doi:10.1371/journal.pone.0319710)
Supplement: S2 Table — (DOCX) [file pone.0319710.s004.docx]

| **S2 Table. Viability of THP-1 cells infected with S and R strains under amikacin and amikacin-free conditions.** | | | | | | | |
| --- | --- | --- | --- | --- | --- | --- | --- |
| **Smooth** | | | | **Rough** | | | **Difference in means** |
| Hours | Mean  Viability(%) | SD ^a^ | CI95% | Mean  Viability(%) | SD ^a^ | CI95% |  |
| 1. **Amikacin-free.** | | | | | | | |
| 2 | 103.9 | 4.9 | 99.9 - 107.9 | 100.6 | 4.4 | 97.2 - 103.9 | 3.34^ns^ |
| 24 | 97.9 | 4.3 | 94.3 - 101.4 | 92.4 | 6.6 | 87.3 - 97.4 | 5.46^ns^ |
| 48 | 74.1 | 7.8 | 67.6 - 80.6 | **65.3** | **7.1** | **59.8 - 70.8** | 8.83* |
| 72 | 62.6 | 6.9 | 56.8 - 68.4 | ***48.4*** | ***6.7*** | ***43.3 - 53.5*** | 14.20** |
| 1. **With amikacin.** | | | | | | | |
| 2 | 98.4 | 4.5 | 94.9 - 102.2 | 100.4 | 38 | 97.5 - 103.3 | -1.98^ns^ |
| 24 | 91.2 | 7.0 | 85.3 - 97.1 | 86.3 | 7.3 | 80.6 - 91.9 | 4.93^ns^ |
| 48 | 79.9 | 7.4 | 73.7 - 86.1 | **75.9** | **8.7** | **69.2 - 82.7** | 4.01^ns^ |
| 72 | 65.5 | 6.1 | 60.5 - 70.6 | ***60.6*** | ***9.4*** | ***53.3 - 67.8*** | 4.97 ^ns^ |
| Statistical analyses presented in this table compare the effects of S and R strains on THP-1 viability under the same condition at each time point. Analyses comparing treatments (amikacin-free vs. amikacin) for each morphotype are not shown in the table but were conducted using the data presented here and are described in the main text. Student's t-test. *p < 0.05.**p<0.001. ^a^ Standard deviation. ^ns^ No significant differences. | | | | | | | |
